# Inference of Gene Regulatory Networks Based on a Universal Minimum Description Length

**DOI:** 10.1155/2008/482090

**Published:** 2008-02-12

**Authors:** John Dougherty, Ioan Tabus, Jaakko Astola

**Affiliations:** 1 Institute of Signal Processing, Tampere University of Technology, P.O. Box 553, 33101 Tampere, Finland

## Abstract

The Boolean network paradigm is a simple and effective way to interpret genomic systems, but discovering the structure of these networks remains a difficult task. The minimum description length (MDL) principle has already been used for inferring genetic regulatory networks from time-series expression data and has proven useful for recovering the directed connections in Boolean networks. However, the existing method uses an ad hoc measure of description length that necessitates a tuning parameter for artificially balancing the model and error costs and, as a result, directly conflicts with the MDL principle's implied universality. In order to surpass this difficulty, we propose a novel MDL-based method in which the description length is a theoretical measure derived from a universal normalized maximum likelihood model. The search space is reduced by applying an implementable analogue of Kolmogorov's structure function. The performance of the proposed method is demonstrated on random synthetic networks, for which it is shown to improve upon previously published network inference algorithms with respect to both speed and accuracy. Finally, it is applied to time-series *Drosophila* gene expression measurements.

## 1. Introduction

The modeling of gene regulatory networks is a major focus of systems biology because, depending on the type of modeling, the networks can be used to model interdependencies between genes, to study the dynamics of the underlying genetic regulation, and to provide a basis for the derivation of optimal intervention strategies. In particular, Bayesian networks [1,2] and dynamic Bayesian networks [3,4] provide models to elucidate dependency relations; functional networks, such as Boolean networks [5] and probabilistic Boolean networks [6], provide the means to characterize steady-state behavior. All of these models are closely related [7].

When inferring a network from data, regardless of the type of network being considered, we are ultimately faced with the difficulty of finding the network configuration that best agrees with the data in question. Inference starts with some framework assumed to be sufficiently complex to capture a set of desired relations and sufficiently simple to be satisfactorily inferred from the data at hand. Many methods have been proposed, for instance, in the design of Bayesian networks [8] and probabilistic Boolean networks [9]. Here we are concerned with Boolean networks, for which a number of methods have been proposed [10–14]. Among the first information-based design algorithms is the Reveal algorithm, which utilizes mutual information to design Boolean networks from time-course data [11]. Information-theoretic design algorithms have also been proposed for non-time-course data [15,16].

Here we take an information-theoretic approach based on the minimum description length (MDL) principle [17]. The MDL principle states that, given a set of data and class of models, one should choose the model providing the shortest encoding of the data. The coding amounts to storing both the network parameters and any deviations of the data from the model, a breakdown that strikes a balance between network precision and complexity. From the perspective of inference, the MDL principle represents a form of complexity regularization, where the intent is generally to measure the goodness of fit as a function of some error and some measure of complexity so as not to overfit the data, the latter being a critical issue when inferring gene networks from limited data. Basically, in addition to choosing an appropriate type, one wishes to select a model most suited for the amount of data. In essence, the MDL principle balances error (deviation from the data) and model complexity by using a cost function consisting of a sum of entropies, one relative to encoding the error and the other relative to encoding the model description [18]. The situation is analogous to that of structural risk minimization in pattern recognition, where the cost function for the classifier is a sum of the resubstitution error of the empirical-error-rule classifier and a function of the VC dimension of the model family [19]. The resubstitution error directly measures the deviation of the model from the data and the VC dimension term penalizes complex models. The difficulties are that one must determine a function of the VC dimension and that the VC dimension is often unknown, so that some approximation, say a bound, must be used. The MDL principle was among the first methods used for gene expression prediction using microarray data [20].

Recently, a time-course-data algorithm, henceforth referred to as Network MDL [10], was proposed based on the MDL principle. The Network MDL algorithm often yields good results, but it does so with an ad hoc coding scheme that requires a user-specified tuning parameter. We will avoid this drawback by achieving a codelength via a normalized maximum likelihood model. In addition, we will improve upon Network MDL's efficiency by applying an analogue of Kolmogorov's structure function [21].

## 2. Background

### 2.1. Boolean Networks

Using notation modified from Akutsu et al. [12], a Boolean network is a directed graph  defined by a set  of  binary-valued nodes representing genes, a collection of structure parameters  indicating their regulatory sets (predecessor genes), and the Boolean functions  regulating their behavior. Specifically, each structure parameter  is the collection of indices  associated with 's regulatory nodes. The number  of regulatory nodes for node  is referred to as the indegree of . We assume that the nodes are observed over  equally spaced time points, and we write  to denote the values of node  for . The value of node  progresses according to(1)

for . Such synchronous updating is perhaps unrealistic in biological systems, but it provides a framework with more easily tractable models and has proven useful in the present context [22]. For ease of notation, we define the inputs of  as the column vector , allowing us to rewrite (1) as(2)

The fundamental question we face is the estimation of  and . Note that  is usually not included as a parameter of  because it can be absorbed into , but we choose to write it separately because, under the model we will specify,  completely dictates , making our interest reside primarily in the structure parameter set .

As written, (2) provides us with a completely deterministic network, but this is generally considered to be an inadequate description. Measurement error is inescapable in virtually any experimental setting, and, even if one could obtain noiseless data, biological systems are constantly under the influence of external factors that might not even be identifiable, let alone measurable [6]. Therefore, we consider it incumbent to relocate our model of the network mechanisms into a probabilistic framework. By incorporating this philosophy and switching to matrix notation, (2) becomes(3)

where  denotes modulo  sum,  acts independently on each column of , and  is a vector of independent Bernoulli random variables with . We further assume that the errors for different nodes are independent. We allow  to depend on  because it can be interpreted as the probability that node  disobeys the network rules, and we consider it natural for different nodes to have varying propensities for misbehaving.

Returning to our overall objective, we observe that  and  can be estimated separately for each gene. This is possible because, for each evaluation of ,  is regarded as fixed and known. Even if a network was constructed so that a gene was entirely self-regulatory, that is, , the random vector  is observed sequentially so that any random variable  within it is observed and then considered as a fixed value  before being used to obtain . Therefore, despite the obvious dependencies that would exist for networks containing configurations such as feedback loops and nodes appearing in multiple predecessor sets, the given model stipulates independence between all random variables. Thus, we restrict ourselves to estimating the parameters for one node and rewrite (3) as(4)

which we recognize as multivariate Boolean regression. Note that  and  now become  and , respectively.

We finalize the specification of our model by extending the parameter space for the error rates by replacing  with  where each  corresponds to one of the  possible values of . This allows the degree of reliability of the network function to vary based upon the state of a gene's predecessors. Note that  is only an upper bound on the number of error rates because we will not necessarily observe all  possible regressor values. This model is specified by the predecessor genes composing , the function , and the error rates in . Thus, adopting notation from Tabus et al. [23], we refer to the collection of all possible parameter settings as the model class 

### 2.2. The MDL Principle

Given the model formulation, we use the MDL principle as our metric for assessing the quality of the parameter estimates. As stated in Section 1, the MDL principle dictates that, given a dataset and some class of possible models, one should choose the model providing the shortest possible encoding of the data. In our case, the MDL principle is applied for selecting each node's predecessors. However, as we have noted, this technique is inherently problematic because no unique manner of codelength evaluation is specified by the principle. Letting  when the node in question is predicted incorrectly and  otherwise, basic coding theory gives us a residual codelength of , but the cost of storing the model parameters has no such standard. Thus, we can technically choose any applicable encoding scheme we like, an allowance that inevitably gives rise to infinitely many model codelengths and, as a result, no unique MDL-based solution.

As an example, we refer to the encoding method used in Network MDL, in which the network is stored via probability tables such as Table 1. In this procedure, the model codelength is calculated as the cost of specifying the two predecessor genes plus the cost of storing the probability table. Letting  and  denote the number of bits needed to encode integers and subunitary floating point numbers, respectively, the model codelength is . Note that we only need  of the probabilities since each row in the table adds to . This is one of many perfectly reasonable coding schemes, but we present another method that corresponds to our model class and yields a shorter codelength. Also, to demonstrate the risk of using the MDL principle with ad hoc encodings, we compare results obtained by using these two schemes in a short artificial example. Observe that Table 1 corresponds to  with each . First, we encode  as the 4 bits  because, providing all predecessor combinations are lexographically sorted, those are the values that  will be with probability . Assuming we select  to minimize the error rates, we can also assume that . Since  bits are sufficient to encode any decimal less than , we really only need  bits to store each , yielding a model cost of .

**Table 1:** Probability table for "OR" function with .

		
	0.8	0.2
	0.2	0.8
	0.2	0.8
	0.2	0.8

To show the effect of the encoding scheme we generated one hundred 6-gene networks, each of which was observed over 50 time points.  and  were fixed so that one gene would behave according to Table 1. The MDL principle was applied for both of the encoding schemes to determine the predecessors of that gene. The results are displayed in Table 2.

**Table 2:** Effect of ad hoc encoding schemes on structure inference. Results are reported as percentages. "Fair" and "Poor" indicate missing one and both of the two predecessors, respectively.

	Encoding method	
Model performance	Network MDL	
Correct	0.03	0.08
Fair	0.12	0.17
Poor	0.85	0.75

We find that the two encoding methods can give different structure estimates because the shorter model codelength allows for a greater number of predecessors. Zhao et al. compensate for this nonuniqueness by adjusting the model codelength with a weight parameter, but, while necessary for ad hoc encodings such as the ones discussed so far, the presence of such tuning parameters is undesirable when compared with a more theoretically based method. Moreover, the MDL principle's notion of "the shortest possible codelength" implies a degree of generality that is violated if we rely upon a user-defined value.

### 2.3. Normalized Maximum Likelihood

One alternative that alleviates these drawbacks is to measure codelength based on universal models. In this approach, we depart from two part description lengths and their ad hoc parameters by evaluating costs using a framework that incorporates distributions over the entire model class. The fundamental idea for such a model is that, assuming a specific model class, we should choose parameters that maximize the probability of the data [21]. Two such models are the mixture universal model and the normalized maximum likelihood (NML) model, the latter of which will command our attention. For  with a fixed , the NML model is introduced by the standard likelihood optimization problem . The solution is obtained for , the maximum likelihood estimate (MLE), but cannot be used as a model because  does not integrate to unity. Thus, we will use the distribution  such that its ideal codelength  is as close as possible to the codelength . This suggests that we should minimize the difference between using  in place of  for the worst case . The resulting optimization problem,(5)

is solved by the NML density function, defined as  divided by the normalizing constant . Tabus et al. [23] provide the derivations of this NML distribution; the following is a brief outline of the major steps.

Given a realization  of the random variable , we have residuals(6)

Recall that the Bernoulli distribution is defined by(7)

Letting  denote the -bit binary representation of integer , combine (6) and (7) to define the probability  as(8)

This representation allows us to formally write our model class as(9)

#### 2.3.1. NML Model for 

Consider any  and fixed . Let  denote the number of times each unique regressor vector  occurs in , and let  count the number of times  is associated with a unitary response. As pointed out by Tabus et al. [23], the MLE for this model is not unique. The network could have , in which case , or , giving . Either way, the NML model is given by(10)

where(11)

Of course, this means that our model does not explicitly estimate . However, considering that  represents error rates, the obvious choice is to minimize each  by taking  whenever , and  otherwise. In the event that , we set  if the portion of  corresponding to  is less than  in binary. Assuming independent errors, this removes any bias that would result from favoring a particular value for  when . This effectively reduces the parameter space for each  from  to  which, in turn, affects  by halving every . However, we will later show that the algorithm does not change whether or not we actually specify , and we opt not to do so.

Also note that computing  exactly may not be feasible. For example, Matlab loses precision for the binomial coefficient  when . In these cases, we use(13)

an approximation given in [24]. For the sake of efficiency, we compute every  prior to learning the network so that calculating the denominator of (10) takes at most  operations.

#### 2.3.2. Stochastic Complexity

We take as the measure of a selected model's total codelength the stochastic complexity of the data, which is defined as the negative base 2 logarithm of the NML density function [21]. As was already the case for the residual codelength, the stochastic complexity is a theoretical codelength and will not necessarily be obtainable in practice, but it is precisely this theoretical basis that frees us from any tuning parameters. Given (10), our stochastic complexity is given by(14)

where  denotes the binary entropy function. Note that the previous and all future logarithms are base 2. Returning to the issue of picking values for , we recall that doing so halves each . This translates to a unit reduction in stochastic complexity for each , but we observe that it also requires  bit to store . Regardless of whether or not we choose to specify , the total codelength remains the same.

The NML model assumes a fixed  to specify the set of predecessor genes, so encoding the network requires that we store this structure parameter as well. The simplest ways to accomplish this are by using  (the total number of genes) bits as indicators or by using  bits to represent the number of predecessors (assuming a uniform prior on ) and  bits to select one of the  possible sets of size . However, the indegrees of genetic networks are generally assumed to be small [25], in light of which we prefer a codelength that favors smaller indegrees and choose to use an upper bound on encoding the integer  to store  with  bits [21]. Note that we use  because the given bound only applies for positive integers, and we must accommodate any . Hence, the total codelength is(15)

where(16)

### 2.4. Kolmogorov's Structure Function

If we compute  for every possible , we can simply select the one that provides the shortest total codelength, thus satisfying the MDL principle; however, this requires computing  codelengths. A standard remedy for this problem is assuming a maximum indegree [12], but, even with , a -gene network would still result in  possible predecessor sets per gene. Moreover, a fixed  introduces bias into the method so, while we obviously cannot afford to perform exhaustive searches, we prefer to refrain from limiting the number of predecessors considered.

Instead, we utilize Kolmogorov's structure function (SF) to avoid excessive computations without sacrificing the ability to identify predecessor sets of arbitrary size. The SF was originally developed within the algorithmic theory of complexity and is noncomputable, so, in order to use this theory for statistical modeling, we need a computable alternative. The details are beyond the scope of this paper, but obtaining a computable SF requires, for fixed , partitioning the parameter space for  so that the Kullback-Leibler distance between any two adjacent partitions, each of which represents a different model, is  for some [21]. When using an NML model class, this partitioning yields an asymptotically uniform prior so that any model  can be encoded with length(17)

where  is the number of error estimates in [21]. Again, the inequality is necessary for data in which not all possible regressor vectors are observed. The partitioning also increases the noise codelength [21] to(18)

We refer to  and  as the model and noise codelengths, respectively, which together constitute a universal sufficient statistics decomposition of the total codelength. The summation of these values is clearly different from the stochastic complexity, but this is a result of partitioning the parameter space.

The appropriate analogue of the SF is then defined as(19)

**Figure 1:**
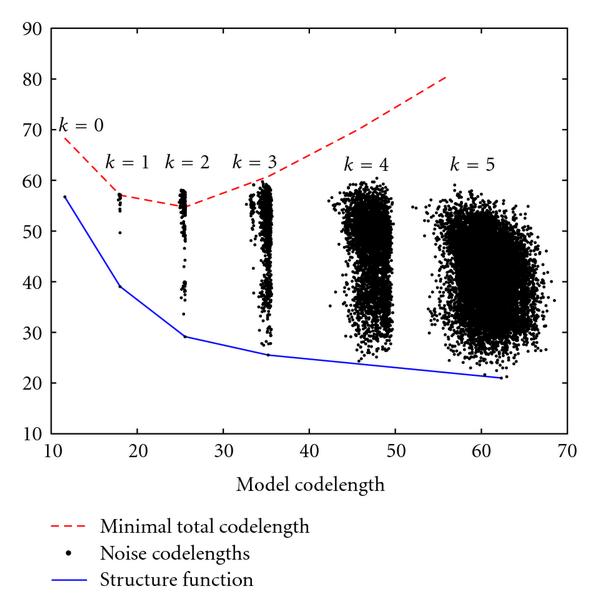
**The SF for a single gene**. The leftmost point is for , and each subsequent vertical band corresponds to a unit increase in . The slope of the SF goes above  after , the same indegree for which the total codelength  is minimized.

We see that  is a nonincreasing function of the model constraint  and displays the minimum possible amount of noise in the data if we restrict the model codelength to be less than . Rissanen shows that this criterion is minimized for [21], but the optimal  cannot be solved analytically. However, by plotting  we obtain a graph similar to a rate-distortion curve (Figure 1), and by making a convex hull we can find a near-optimal predecessor set. Simply select the truncation point at which the magnitude of the slope of the hull drops below . In other words, locate the truncation point at which allowing an additional bit for the model yields less than a -bit reduction in the noise codelength because, once past this point, increasing the model complexity no longer decreases the total encoding cost.

Of particular use in this scenario is the way in which the model codelength is somewhat stable for each , producing the distinct bands in Figure 1. The noise codelengths are still widely dispersed so we are required to compute all possible codelengths up to some total number of predecessors. We would like that number to be variable and not arbitrarily specified in advance, but this may not be feasible for highly connected networks. However, as mentioned earlier, the indegrees of genetic networks are generally assumed to be small (hence, the standard ), and, when looking for a single gene's predecessors in a 20-gene network, our method only takes 70 minutes to check every possible set up to size 6. Thus, we are still constrained by a maximum indegree, but we can now increase it well beyond the accepted number that we expect to encounter in practice without risking extreme computational repercussions. Additionally, choosing a  makes  a nondecreasing function of , meaning that we can also stop searching if  ever becomes larger than the current value of . The method is summarized in Algorithm 1.

**Algorithm 1:** The NML MDL method for one gene.

(1) Initialize 

(2) 

(3) 

(4) **for** to **do**

(5)* * compute  using (16)

(6)* ***if****then**

(7)*  ***return**

(8)* ***end if**

(9)  collection of all 's such that 

(10)* ***for** to **do**

(11)*  * rows of  specified by 

(12)*  ***for** to **do**

(13)*   * compute  and  for 

(14)*  ***end for**

(15)  number of nonzero 's

(16)*  * compute  and 

*   *       using (11), (17), and (18)

(17)* ***end for**

(18)* * use , , , and  to form a convex

*  *     hull with truncation points 

 (19) 

(20)* ***if** isempty () **then**

(21)*  ***return**

(22)* ***else**

(23)*  * update , and  using truncation

*   *     point indexed by 

(24)* ***end if**

(25) **end for**

Note that we termed the resulting predecessors "near-optimal." It is possible to encounter genes for which adding one predecessor does not warrant an increase in model codelength but adding two predecessors does. Nevertheless, these differences tend to be small for certain types of networks. Moreover, depending on the kind of error with which one is concerned, these near-optimal predecessor sets can even provide a better approximation of the true network in the sense that any differences will be in the direction of the SF finding fewer predecessors. Thus, assuming a maximum indegree , the false positive rate from using the SF can never be higher than that from checking all predecessor sets up to size .

## 3. Results

### 3.1. Performance on Simulated Data

A critical issue in performance analysis concerns the class from which the random networks are to be generated. While it might first appear that one should generate networks using the class  composed of all Boolean networks containing  genes, this is not necessarily the case if one wishes to achieve simulated results that reflect algorithm performance on realistic networks. An obvious constraint is to limit the indegree, either for biological reasons [26] or for the sake of inference accuracy when data are limited. In this case, one can consider the class  composed of all Boolean networks with indegrees bounded by . Other constraints might include realistic attractor structures [27], networks that are neither too sensitive nor too insensitive to perturbations [28], or networks that are neither too chaotic nor too ordered [29].

Here we consider a constraint on the functions that is known to prevent chaotic behavior [5,26]. A canalizing function is one for which there exists a gene among its regulatory set such that if the gene takes on a certain value, then that value determines the value of the function irrespective of the values of the other regulatory genes. For example,  OR  is canalizing with respect to  because  for any values of  and . There is evidence that genetic networks under the Boolean model favor this kind of functionality [30]. Corresponding to class  is class , in which all functions are constrained to be canalizing.

To evaluate the performance of our model selection method, referred to as NML MDL, on synthetic Boolean networks, we consider sample sizes ranging from  to , , and . We test each of the  combinations on  randomly generated networks from  and . Note that  is equivalent to .

We use the Reveal and Network MDL methods as benchmarks for comparison. As mentioned earlier, Network MDL requires a tuning parameter, which we set to  since that paper uses 0.2–0.4 as the range for this parameter in its simulations. Also, its application in [10] limits the average indegree of the inferred network to 3 so we assume this as well. Reveal is run from a Matlab toolbox created by Kevin Murphy, available for download at http://bnt.sourceforge.net/, and requires a fixed , which we also set to 3. We implement our method with and without including the SF approach to show that the difference in accuracy is often small, especially in light of the reduction in computation time.

As performance metrics, we use the number of false positives and the Hamming distance between the estimated and true networks, both normalized over the total number of edges in the true network. False positives are defined as any time a proposed network includes an edge not existing in the real network, and Hamming distance is defined as the number of false positives plus the number of edges in the true network not included in the estimated network.

#### 3.1.1. Random Networks

**Figure 2:**
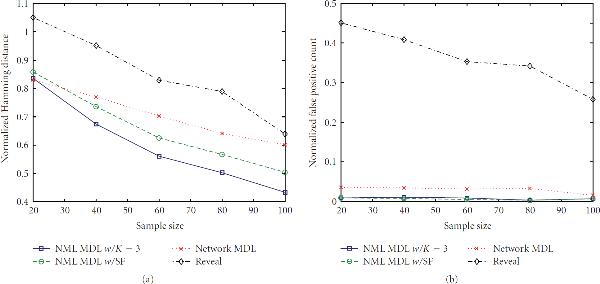
**(a) Hamming distances and (b) false positive counts for random networks generated from  with **. Results are normalized over the true number of connections and averaged over 30 networks.

**Figure 3:**
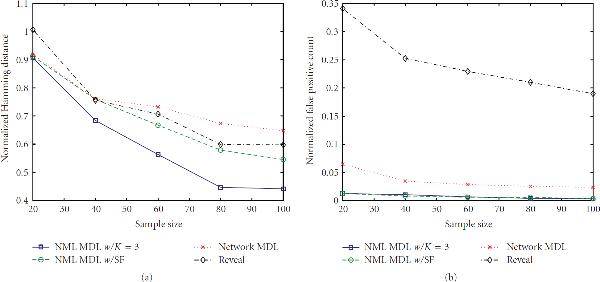
**Error rates for  and **.

**Figure 4:**
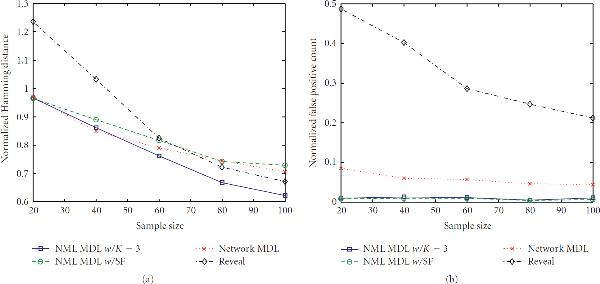
**Error rates for  and **.

**Figure 5:**
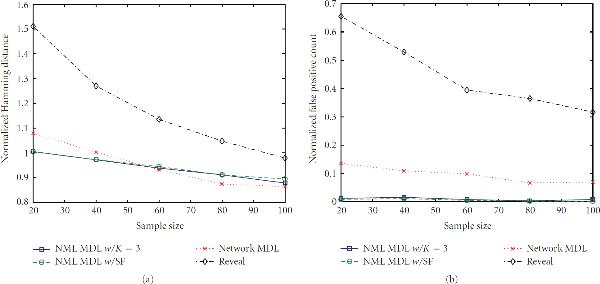
**Error rates for  and **.

In this section, we consider performance when the network is generated from . Figures 2–5 show a selection of the performance-metric results for all four methods and several combinations of  and . The remaining figures can be found in the supporting data, available at http://www.stat.tamu.edu/~jdougherty/nmlmdl.

With respect to false positives, NML MDL is uniformly the best, and there is at most a minor difference between the two modes. NML MDL is also the best overall method when looking at Hamming distances. Figures 2 and 3 show the cases for which it most definitively improves upon Network MDL and Reveal, both of which have . The way in which the two NML methods diverge as  increases is a general trend, but both remain below Network MDL. Increasing  to 0.2 narrows the margins between the methods, but the relationships only change significantly for . As shown in Figure 4, NML MDL with the SF loses its edge, but NML MDL with fixed  remains the best choice. Raising  to 0.3 is most detrimental to Reveal, pulling its accuracy well away from the other three methods. Figure 5 shows this for , but the plots for smaller values of  look very similar, especially in how the two NML MDL approaches perform almost identically. We point out that this is the worst scenario for NML MDL, but, even then, it is still superior for small  and only worse than Network MDL for .

In terms of computation time, Reveal was fairly constant for all of the simulation settings, taking an average of 6.35 seconds to find predecessors for gene using Matlab on a Pentium IV desktop computer with 1 GB of memory. NML MDL with  increases slightly with  in a linear fashion, but its most noticeable increase is with . For , this method took an average of 0.33 to 0.48 seconds per gene as  goes from 20 to 100, but this range increased from 0.59 to 0.73 for . Alternatively, Network MDL's runtime is sporadic with respect to  and decreases when  is raised, taking an average of 2.50 seconds per gene for  but needing only 0.33 second per gene when , the only case for which it was noticeably faster than NML MDL with fixed . However, NML MDL with the SF proved to be the most efficient algorithm in almost every scenario. For  and 0.3 it was uniformly the fastest, taking an average of 0.06 and 0.02 seconds per gene, respectively. The runtime begins to increase more rapidly with  for  and , but the only observed case when it was not the fastest method was for  and , and even then the needed time was still less than 1 second per gene.

#### 3.1.2. Canalizing Networks

**Figure 6:**
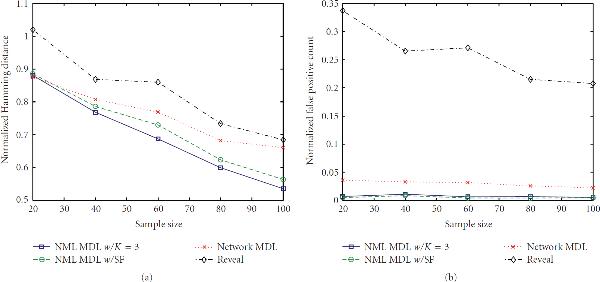
**Error rates for  and **.

Next, we impose the canalizing restriction and generate networks from . The general impact can be seen by comparing Figures 3 and 6. There is essentially no difference in the false positive rates (or runtimes), but the behavior of the Hamming distances is clearly different. We observe that NML MDL with fixed  performs better over all Boolean functions, although invoking the SF yields error rates much closer to the fixed  approach when we are restricted to canalizing functions. This is expected because one canalizing gene can provide a significant amount of predictive power, whereas a noncanalizing function may require multiple predecessors to achieve any amount of predictability.

For example, consider  OR . If  is found to be the best predecessor set of size 1, adding  may not give enough additional information to warrant the increased model codelength, in which case NML MDL will miss one connection. Alternatively, if  XOR , either input tells almost nothing by itself, and the SF will probably stop the inference too soon. However, using both inputs will most likely result in the minimum total codelength, in which case NML MDL with fixed  will find the correct predecessor set.

For the same reason, we also see that Network MDL is better suited to canalizing functions, but Reveal does better without this constraint. Of particular interest is that, for these methods, the change can be so drastic that they comparatively switch their rankings depending on which network class we use, whereas NML MDL provides the most accurate inference either way. Similar results can be observed for the other cases in the supporting data. Based on these findings, we recommend using the SF primarily for networks composed of canalizing functions and networks too large to run NML MDL with fixed  in a reasonable amount of time. We also suggest using the SF when  is large because, as pointed out in Section 3.1.1, the performance of the two NML MDL varieties is no longer different when .

### 3.2. Application to Drosophila Data

**Figure 7:**
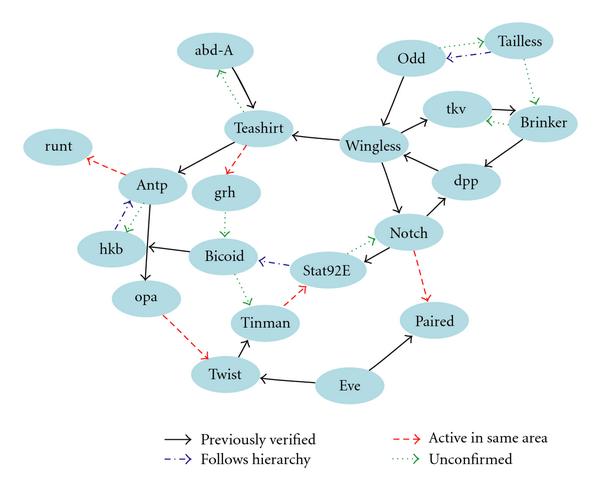
**Inferred gene regulatory network for *Drosophila***.

In order to examine the proficiency of NML MDL on real data, we tested it on time-series Drosophila gene expression measurements made by Arbeitman et al. [31]. The dataset in question consists of 4028 genes observed over 67 time points, which we binarized according to the procedure outlined in [10]. We selected 20 of these genes based on type (gap, pair-rule, etc.) and the availability of genetically verified directed interactions in the literature. Of the 32 edges identified by NML MDL (Figure 7), 16 have been previously demonstrated [32–43], and 3 more follow the standard genetic hierarchy [44]. Observe that 3 of the 12 other edges are simply reversals of known relationships and, therefore, could possibly represent unknown feedback mechanisms. Additionally, 5 of the remaining inferred relationships are between genes that are active in the same area such as the central nervous system (*Antp*/*runt*) and reproductive organs (*Notch*/*paired*) (the Interactive Fly website, hosted by the Society for Developmental Biology).

## 4. Concluding Remarks

Using a universal codelength when applying the MDL principle eliminates the relativity of applying ad hoc codelengths and user-defined tuning parameters. In our case, this has resulted in improved accuracy of Boolean network esimation. Using the theoretically grounded stochastic complexity instead of ad hoc encodings genuinely reflects the intent of the MDL principle. In addition, the structure function makes the proposed method faster than other published methods. Computation time does not heavily rely on bounded indegrees and increases only slightly with .
